# Effect of Rare Earth Elements (La and Ce) on Microstructure and Mechanical Properties of U75V Steel

**DOI:** 10.3390/ma19020370

**Published:** 2026-01-16

**Authors:** Mengqiang Hu, Lei Ren, Guangqian Feng, Jichun Yang, Yubao Liu

**Affiliations:** 1 School of Rare Earth Industry, Inner Mongolia University of Science and Technology (IMUST), Baotou 014010, China; 19806101107@163.com (M.H.); yangjichun1963@163.com (J.Y.); 2 Shaanxi Changyu Aviation Assembly Co., Ltd., Xi’an 710016, China; 3 State Key Laboratory of Baiyunobo Rare Earth Resource Researches and Comprehensive Utilization, Baotou Research Institute of Rare Earths, Baotou 014030, China

**Keywords:** multiple-element rare earths, U75V steel, inclusions, microstructure, mechanical properties

## Abstract

To investigate the effects of multiple-element rare earth addition on U75V steel, this study produced three types of steel: sample 1 steel without rare earths, sample 2 steel containing 0.0035% La and 0.018% Ce, and sample 3 steel containing 0.02% La and 0.0023% Ce. Microstructural analysis showed that the addition of rare earth elements modified the MnS and silicoaluminate inclusions into RE_2_O_2_S and RE_2_O_2_S–oxide complexes, which reduced the number and size of inclusions while simultaneously refining the microstructure, including the grain size and the spacing of pearlite layers. Concurrently, RE addition enhanced the steel’s mechanical properties, with the degree of enhancement dependent on RE content; sample 2 exhibited the most balanced improvement. Compared to sample 1, the hardness of samples 2 and 3 increased by 15.3% and 3.6%, respectively, and their tensile strength increased by 7.9% and 6.8%, respectively. Meanwhile, their coefficients of friction decreased significantly, by 69.5% and 22.1%. The impact toughness was also enhanced by RE addition, with both samples 2 and 3 showing higher values than sample 1 at room temperature and moderate low temperatures. Nevertheless, a distinct reversal was observed at −60 °C, where the impact energy of sample 3 was 23.5% lower than that of sample 2. This result implies that while moderate RE addition is beneficial, an excessive amount can adversely affect the toughness under cryogenic conditions.

## 1. Introduction

Railway transportation plays a critical role in modern transport systems. The rail, as a key component of the track structure, directly influences the safety, stability, and service life of railway operations [1,2,3,4]. U75V steel, a widely used high-strength rail steel for Chinese mainlines, offers excellent comprehensive mechanical properties and withstands complex loading during train operations [5]. However, the continuous development of railways towards higher speeds and heavier loads imposes increasingly stringent demands on rail performance. Consequently, enhancing the properties of U75V steel has become an active research area in steel materials. Due to their unique outer electronic structure and physicochemical properties, rare earth elements show great potential in microalloying of steel materials. Among them, La and Ce, as the more common rare earth elements with relatively abundant reserves, have attracted much attention in improving the organization and properties of metallic materials. Numerous studies have shown [6,7,8] that trace amounts of rare earth elements added to steel can play a positive role in purifying the steel, denaturing inclusions, and refining grain size. However, their contents have a great influence on the properties of steel. Guo et al. [9] investigated the effects of Ce addition on MnS inclusions in U75V rail steel. Ce spheroidized and dispersed MnS inclusions, with optimal refinement (minimum average size) occurring at 139 ppm Ce content, thereby enhancing mechanical properties. Kang et al. [10] demonstrated that adding 0.0066% rare earth elements (REEs) effectively reduces sulfur content and eliminates elongated MnS inclusions along with large irregular Al-Mn-O inclusions. These are modified into fine spherical REAlO_3_-MnS duplex inclusions, resulting in microstructural refinement and markedly enhanced low-temperature impact toughness. Guo et al. [11] investigated the effects of varying La additions on 3Cr2W8V hot-work die steel. La within the optimal range significantly refined the microstructure and enhanced strength, impact toughness, and ductility. Peak comprehensive mechanical properties were achieved at 0.20% La addition. Jie et al. [12] demonstrated that rare earth elements significantly enhance the thermal fatigue resistance of 5CrMnMo steel. Maximum improvement occurred at 0.05% residual REE content. Beyond this threshold, RE-bearing inclusions increased, causing property deterioration.

Furthermore, REEs effectively regulate the morphology, size, and distribution of inclusions in steels [13]. Shi et al. [14] demonstrated that increasing Ce content in 4Cr5MoSiV1 steel reduces inclusion density and average size while modifying inclusions into spherical RE–sulfoxides. Minimum inclusion density and finest average size occurred at 0.0070% Ce. Fan Lei et al. [15] found that in high-sulfur free-cutting steel, MnS can precipitate on the surface of CeAlO_3_ to form composite inclusions when the Ce content is 0.01%, and MnS can wrap with Ce_2_S_3_ to form composite inclusions when the Ce content is 0.05% and the size of sulfide is reduced. Ma Haitao et al. [16] showed that the addition of rare earths in BG420CL wheel steel can change the shape and size of inclusions, and the spheroidization of inclusions is best when the amount of rare earths added is about RE/S = 2.0. After the addition of lanthanum and cerium to heavy rail steel, most of the lanthanum and cerium elements are biased at grain boundaries through diffusion mechanisms and combine with oxygen, sulfur and other elements to form compounds such as rare earth oxides, rare earth sulfides and other compounds, which replace the position of impurity elements at grain boundaries and strengthen the grain boundaries [17].

For U75V steel, introducing multi-component rare earth elements (La and Ce) enables refined microstructural control to further enhance mechanical properties, meeting the stringent demands of modern railway transportation. Although prior studies have examined REE effects in other steels [18,19,20,21,22], systematic investigations on La-Ce-modified U75V steel remain limited, particularly regarding microstructural evolution and its correlation with mechanical performance [23]. This study, therefore, systematically investigates the effects of varying La-Ce additions on U75V steel’s microstructural characteristics and mechanical properties (tensile strength and impact toughness). The underlying mechanisms of multi-REEs are elucidated to advance performance optimization in rail-grade steels.

## 2. Materials and Methods

### 2.1. Preparation of U75V Steel

**Figure 1:**
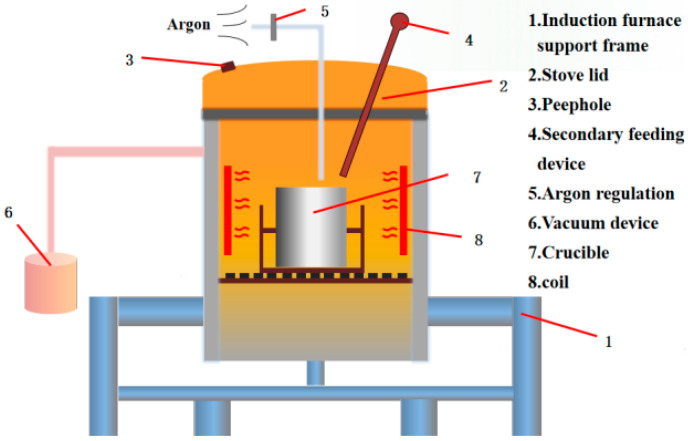
Schematic diagram of a vacuum induction furnace.

A 10 kg vacuum induction furnace (Model ZG 0.01 from Xi‘an North Gaosheng Electric Furnace Equipment Co., Ltd., Xi’an, China) was employed in this study, with its technical parameters and a schematic diagram provided in Table 1 and Figure 1, respectively. Prior to smelting, the furnace was prepared by placing an asbestos mesh within the induction coil and ramming a mixture of magnesia sand and water glass into the gap between an MgO crucible and the mesh. An iron bar was then charged into the crucible and heated until the lining was completely dry.

The smelting process proceeded as follows: (1) Pure iron was loaded into the crucible, and the furnace was evacuated to 15 Pa. (2) The power was set to 40 kW to melt the pure iron. (3) After complete melting, the power was reduced to 20 kW for a 15 min refining period under an argon atmosphere. (4) Alloying elements (carbon powder, crystalline silicon, manganese metal, ferrovanadium, lanthanum, and cerium) were added sequentially. (5) Following complete melting and homogenization, the melt was poured, cooled, and the final ingot was discharged.

Three independent melts of U75V steel were produced for this investigation. Following the removal of shrinkage cavities from the ingots, the chemical composition was analyzed on samples taken from the sound sections. The concentrations of key elements (C, Si, Mn, and V) were determined directly using a Labspark-1000 optical emission spectrometer (from Suzhou Gangyan Nake Testing Technology Co., Ltd., Suzhou, China). In contrast, the trace levels of oxygen (O), lanthanum (La), and cerium (Ce) were measured by an external agency (Steel Research Institute) to ensure accuracy. As summarized in Table 2, the base compositions of all three experimental steels met the national standard for U75V grade. The specific rare earth additions defined the three distinct samples: sample 1 (RE-free), sample 2 (0.0035% La and 0.018% Ce), and sample 3 (0.02% La and 0.0023% Ce).

### 2.2. Microstructure Observation

**Figure 2:**
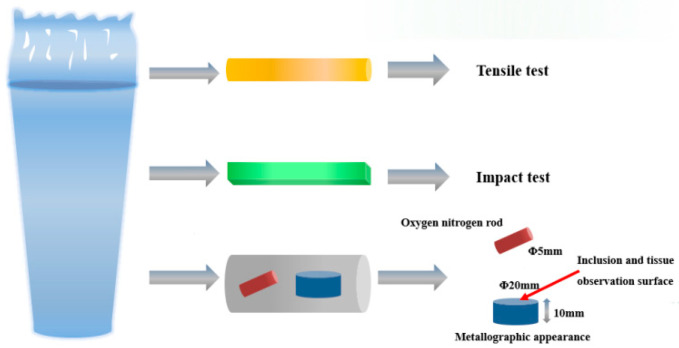
Schematic diagram of sampling and processing of experimental steel.

Specimens with dimensions of φ20 mm × 10 mm were sectioned using a molybdenum wire cutting machine (from Laitesi, Taizhou, China). They were then ground sequentially using 400# to 2000# grit sandpaper and subsequently polished on a metallographic polishing machine (from Shanghai Senbo New Materials Technology Co., Ltd., Shanghai, China) to achieve a smooth, scratch-free surface. The polished specimens were etched with a 4% nital solution for 5–8 s until a light haze appeared, followed by rinsing with alcohol and drying under a warm air stream. Microstructural characterization and inclusion analysis were performed using a ZEISS Sigma 300 ultra-high-resolution scanning electron microscope (SEM from Carl Zeiss AG, Jena, Germany) equipped with an Oxford X-MaxN20 energy-dispersive spectrometer EDS (from Suzhou Science Instrument Co., Ltd., Suzhou, China). A schematic illustrating the sample preparation procedure is provided in Figure 2.

### 2.3. Mechanical Performance Test

**Figure 3:**
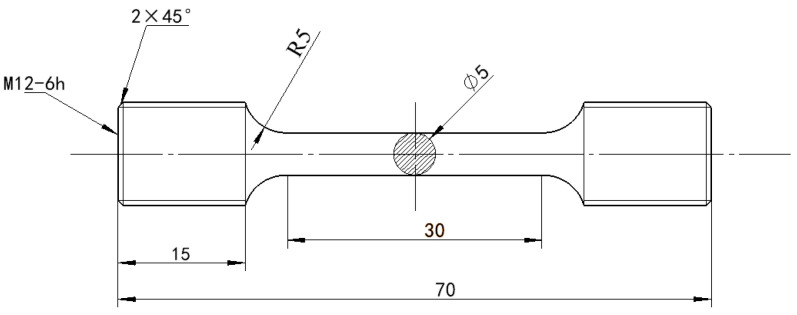
Schematic diagram of a tensile specimen.

**Figure 4:**
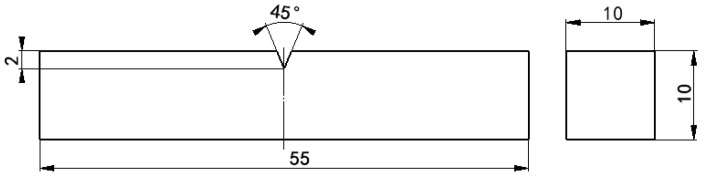
Schematic diagram of the impact sample.

The tensile specimen was machined to the dimensions illustrated in Figure 3, and the tensile test was conducted on a GNT universal testing machine (from Suzhou Gangyan Nake Testing Technology Co., Ltd., Suzhou, China). In accordance with the GB/T229-1994 standard [13], the impact specimens were machined longitudinally to dimensions of 10 mm × 10 mm × 55 mm, as shown in Figure 4. Charpy impact tests at room temperature were performed using a pendulum impact tester (from Shenglin Precision Machinery Equipment, Jinan, China). The hardness was measured using a microhardness tester (from Shenzhen Haoxinda Instrument Co., Ltd., Shenzhen, China). To minimize experimental error, the specimen surface was polished to a smooth finish, and ten indentation measurements were taken and averaged for each sample.

## 3. Results and Discussion

### 3.1. Effect of Rare Earths La and Ce on the Microstructure of U75V Steel

#### 3.1.1. Effect of Rare Earths La and Ce on the Composition and Morphology of Inclusions in U75V Steel

**Figure 5:**
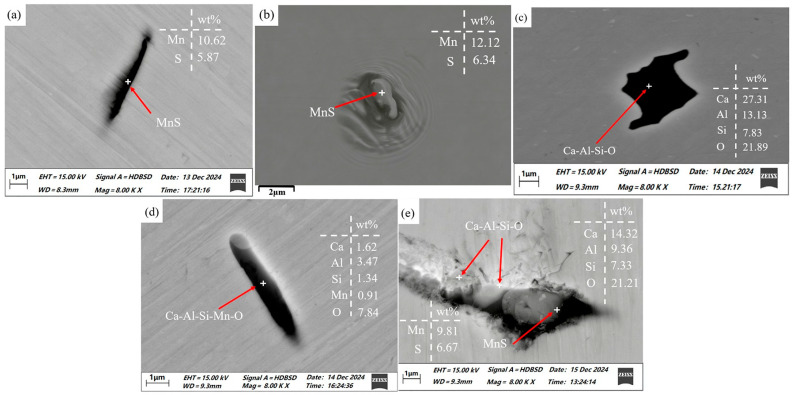
MnS and oxide inclusions in sample 1 without rare earth additives: (**a**) MnS inclusions; (**b**) MnS inclusions under secondary electrons; (**c**) Ca-Al-Si-O inclusions; (**d**) Ca-Al-Si-Mn-O inclusions; (**e**) Ca-Al-Si-O and MnS composite inclusions.

Observations and inclusion counts were conducted using a ZEISS Sigma 300 scanning electron microscope. The inclusions in sample 1 (without rare earth addition) are categorized as follows. The first category consists of MnS inclusions, most of which exhibit an elongated, strip-like morphology, as shown in Figure 5a. The presence of low-strength voids around these inclusions causes the surrounding steel matrix to be pulled out during the grinding and polishing process, as shown in Figure 5b. MnS inclusions possess the highest linear expansion coefficient among all common inclusions in steel, which differs significantly from that of the steel matrix. Consequently, they shrink rapidly during cooling, promoting the formation of surrounding voids. These voids readily become initiation sites for fatigue cracks [24]. Furthermore, MnS inclusions are typical plastic inclusions with a low melting point. During rolling, they are easily elongated along the rolling direction. Subsequently, when the steel is subjected to external forces in service, the deformation of the steel matrix causes the voids associated with these elongated inclusions to grow. This can lead to crack formation within the steel or contribute to property anisotropy, ultimately shortening the service life of the material [25].

The second type of inclusions is oxide inclusions, as shown in Figure 5c,d. Such complex oxides are common in U75V steels and exhibit highly irregular shapes with sharp angles. These oxide inclusions are generally large. Furthermore, the significant difference between their thermal expansion coefficient and that of the steel matrix generates local stress during heat treatment. During service, this residual stress superimposes with the applied load. Additionally, due to their high hardness, these oxide inclusions cannot undergo coordinated plastic deformation with the matrix. This incompatibility leads to stress concentration at the inclusion/matrix interface, facilitating the initiation of micro-voids or cracks. These defects severely deteriorate the toughness and fatigue properties of the steel.

The third type of inclusion comprises composite inclusions of oxides and MnS, which typically exhibit an overall spindle-shaped morphology, as shown in Figure 5e. Since MnS inclusions precipitate at a lower temperature than (Ca, Al, Si, Mn)-based oxides, they tend to nucleate and grow on the pre-existing high-melting-point oxide cores during solidification, forming duplex inclusions with a core–shell structure. These composite inclusions often exhibit irregular shapes and are generally larger in size. This type of composite inclusion is notably detrimental to the properties of U75V steel. On the one hand, the ductile MnS shell tends to elongate along the rolling direction during processing, forming a “soft shell–hard core” structure with the rigid oxide core inside. This markedly weakens the inclusion–matrix interfacial bonding strength. On the other hand, the irregular morphology of composite inclusions (e.g., angular or elongated shapes) acts as stress concentration points within the steel matrix [6,7].

**Figure 6:**
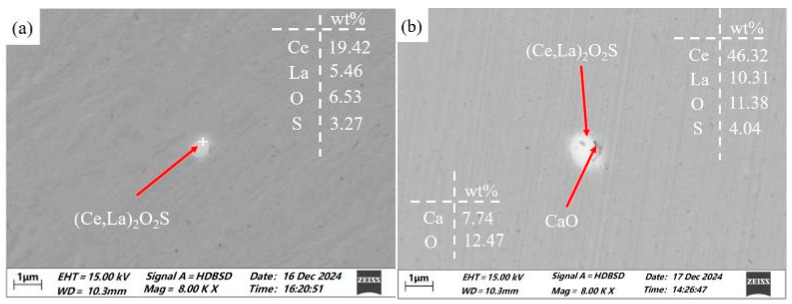
Pure rare earth inclusions and composite inclusions in steel containing mixed rare earths: (**a**) (Ce, La)_2_O_2_S inclusions; (**b**) (Ce, La)_2_O_2_S-CaO composite inclusions.

The types of inclusions found in the RE-added steels (samples 2 and 3) are similar. The first category is pure rare earth inclusions, as shown in Figure 6a. In the backscattered electron images, these rare earth inclusions appear bright white and were primarily composed of (Ce, La)_2_O_2_S, with a small number of (Ce, La)_x_S_y_ compounds. Overall, these pure rare earth inclusions are not only relatively small in size but also tend to be spherical. The second type consists of composite inclusions containing both rare earth elements and oxides, as shown in Figure 6b. Although larger than the pure rare earth inclusions, these composite particles still maintain a relatively regular shape.

To elucidate the formation mechanism and spherical morphology of RE_2_O_2_S, a thermodynamic analysis was conducted based on existing literature data and experimental compositional characteristics. From a thermodynamic perspective, compounds with a lower standard Gibbs free energy of formation exhibit higher thermodynamic stability and are more readily formed during reactions. According to thermodynamic data reported by Hao et al. [26] and Feng et al. [27], the standard Gibbs free energy of formation for rare earth oxysulfides (RE_2_O_2_S) is significantly lower than that of manganese sulfide (MnS) and various calcium–aluminosilicate oxide inclusions. This indicates that rare earth elements (La and Ce) possess an exceptionally strong affinity for oxygen and sulfur in molten steel, enabling them to combine with dissolved [O] and [S] before other elements can do so. As a result, highly stable RE_2_O_2_S phases form, which thermodynamically favor the transformation of the original inclusions rather than the preservation of their initial composition and morphology.

Regarding morphological evolution, the RE_2_O_2_S phase typically adopts a highly symmetrical hexagonal crystal structure. Its surface energy in molten steel is considerably lower than that of elongated MnS and angular oxide inclusions [28,29]. Following thermodynamic principles, during solidification, inclusions tend spontaneously to adopt low-surface-energy morphologies to minimize the total Gibbs free energy of the system. Consequently, RE_2_O_2_S and its composite inclusions assume a spherical or near-spherical shape.

#### 3.1.2. Effect of Rare Earths La and Ce on the Number and Size of Inclusions in U75V Steel

**Figure 7:**
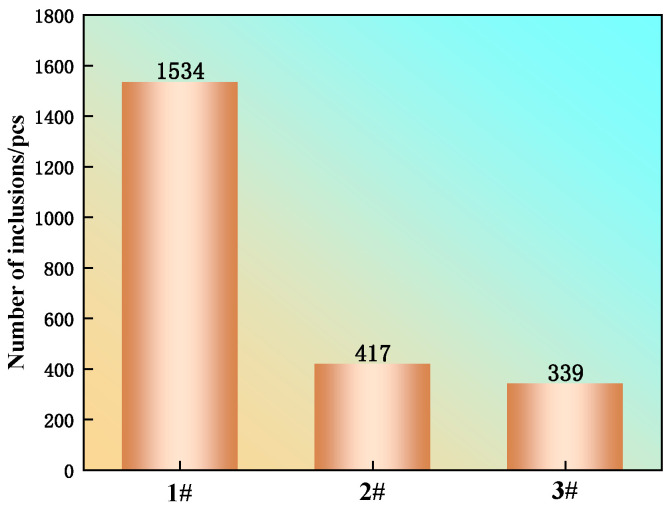
Changes in the number of inclusions in steel before and after adding multi-element rare earths.

The number and size of inclusions in the steel were statistically analyzed using a ZEISS Sigma 300 scanning electron microscope equipped with an automatic inclusion analyzer. The analysis was performed over a scanning area of 4.45 mm^2^ at a magnification of 600. The statistical results (Figure 7) revealed that the total inclusion count in sample 1 was 1534. In contrast, the counts in samples 2 and 3, with the addition of multi-component rare earths, were significantly reduced to 417 and 339, respectively. This corresponds to a reduction of 72.8% and 77.7% compared to sample 1.

Zhang et al. [30] conducted three-dimensional morphology observations of MnS inclusions in heavy rail steel casting billets. They reported that these MnS inclusions exhibited spherical, spindle-like, elongated, and plate-like morphologies and readily combined with oxide inclusions to form new composite inclusions. The sharp decrease in inclusion number after rare earth addition is attributed to the higher content of multi-component rare earths, which leads to a more pronounced modification effect on the original inclusions in the steel. Upon addition, the rare earth elements rapidly react with O and S to form numerous fine rare earth oxides and oxysulfides. These newly formed particles can further combine with existing inclusions, such as CaO, to generate rare-earth-based composite inclusions. These types of inclusions possess high melting points. Furthermore, rare earth inclusions have high surface activity, making the fine particles more likely to coalesce and grow during the solidification of steel. Consequently, most of these agglomerated inclusions can be removed from the melt by flotation.

**Figure 8:**
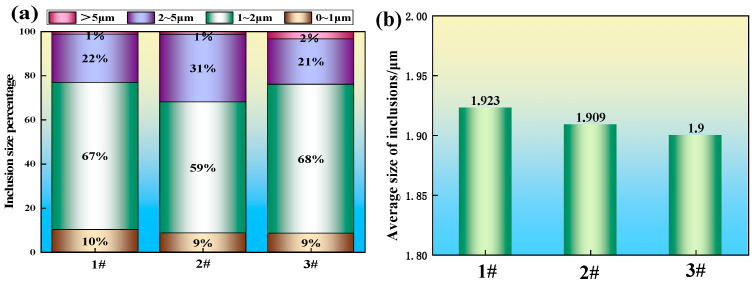
Percentage and average size of inclusions in steel: (**a**) percentage of inclusion size; (**b**) average size of inclusions.

As shown in Figure 8a, the inclusions in all three experimental steels are predominantly smaller than 5 μm, with those under 2 μm being the most prevalent, which is consistent with the findings of Bai Guojun et al. [31]. For sample 2 with multi-component rare earth addition, the proportion of inclusions smaller than 2 μm decreased, while the fraction in the 2−5 μm range increased. A similar trend was observed in sample 3, where the percentage of inclusions smaller than 1 μm decreased, and the proportion in the 2−5 μm range increased; however, the fraction of inclusions larger than 5 μm also showed a slight increase in this sample. Although the proportion of large-sized inclusions increased slightly in steels 2 and 3, their negative impact on steel purity was limited because the rare earth addition significantly reduced the overall number of inclusions (see Figure 7). As presented in Figure 8b, the average inclusion sizes for samples 1, 2, and 3 are 1.923 μm, 1.909 μm, and 1.900 μm, respectively. This indicates a slight refinement in average inclusion size following the addition of rare earths. Considering the changes in both size distribution and total number, although some large-sized inclusions persisted, the average size was not significantly elevated due to the reduction in their absolute quantity. This demonstrates that rare earth elements are effective in purifying the steel by refining inclusions.

#### 3.1.3. Effect of Rare Earths La and Ce on the Grain Size of U75V Steel

**Figure 9:**
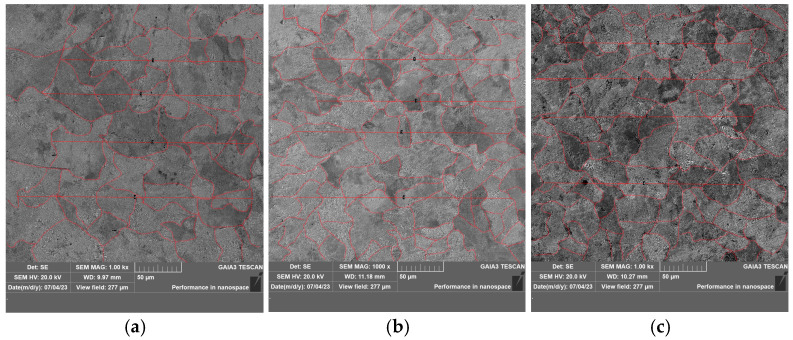
Micrograph of grain structure in U75V experimental steel: (**a**) sample 1; (**b**) sample 2; (**c**) sample 3.

**Figure 10:**
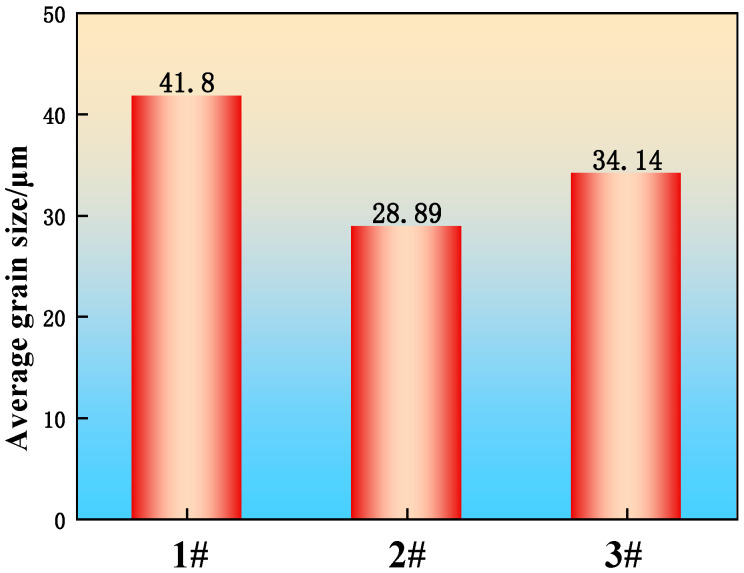
Average grain size statistics.

The sample grains were observed and analyzed using a scanning electron microscope, and the results are shown in Figure 9. The grain size was measured from these images using Image software (version 2.16) via the linear intercept method. To ensure accuracy, ten measurements were taken in each of eight randomly selected fields of view per steel, and the results were averaged. The resulting grain size statistics are presented in Figure 10.

The average grain size in sample 1 without added rare earths is 41.8 μm, and the average grain size in samples 2 and 3 with added poly-rare earths is 28.89 μm and 34.14 μm, which are reduced by 30.1% and 18.3%, respectively. Compared with the RE-free steel, the RE-added steels exhibited a finer and more uniform grain size distribution. This refinement is primarily attributed to the formation of numerous, finely dispersed rare earth inclusions with high melting points upon addition to the liquid steel. The fine rare earth oxysulfides and sulfides in samples 2 and 3 exhibited a low lattice mismatch with δ-ferrite, enabling them to act effectively as heterogeneous nucleation sites [32]. The addition of rare earth elements mitigates the segregation of impurity elements such as S and P at the grain boundaries, thereby purifying the boundaries. Additionally, the rare earth elements themselves tend to segregate at the grain boundaries, which reduces the interfacial tension and energy, consequently lowering the driving force for grain growth and enhancing the boundary strength. These combined effects contribute to the overall grain refinement [33].

**Figure 11:**
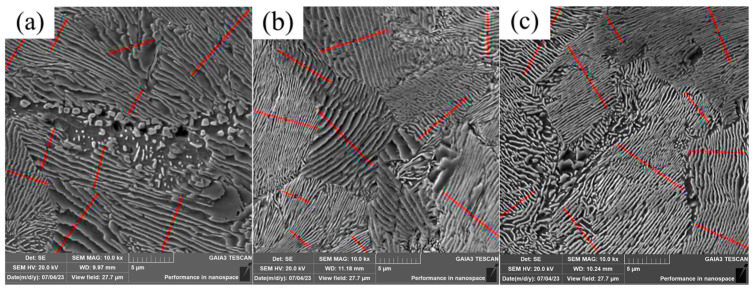
Pearlite lamellae in U75V experimental steel: (**a**) sample 1; (**b**) sample 2; (**c**) sample 3.

**Figure 12:**
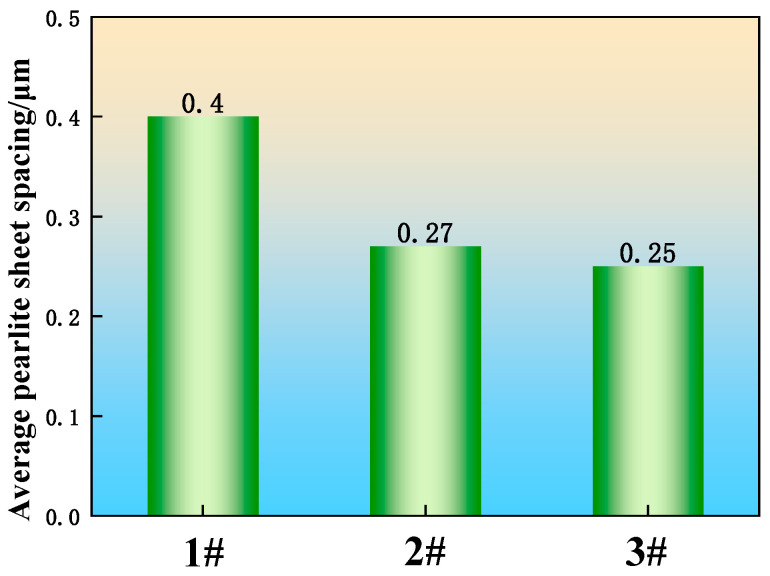
Average pearlite sheet spacing for U75V steel.

The field emission scanning electron microscope was used to observe the U75V experimental steel pearlite, and the Image software was used to measure the pearlite lamellae, and the measurement method was the truncation method, as shown in Figure 11. To ensure the accuracy of the experiment, each experimental steel selected 8 fields of view for statistics, each field of view measured 10 groups, and the measurement results took the average value; the measurement results are shown in Figure 12. The average pearlite lamellae layer of sample 1 without added rare earths is 0.40 μm, and the average pearlite lamellae layer of samples 2 and 3 with added poly-rare earths is 0.27 μm and 0.25 μm, respectively, which is a reduction of 32.5% and 37.5%, respectively, compared with that of sample 1 without added rare earths.

Pearlite is a lamellar microstructure composed of alternating layers of ferrite and cementite (carbide). The atomic radii of lanthanum (La) and cerium (Ce) are larger than that of iron (Fe). Their dissolution in ferrite introduces significant lattice distortion energy, which promotes their segregation to the ferrite–cementite interface. The relationship for the pearlite interlamellar spacing is given by Equation (1). This equation indicates that the addition of rare earth elements reduces the interfacial energy between ferrite and cementite, thereby leading to a finer pearlite interlamellar spacing. The pearlitic transformation is a diffusion-controlled process. The reduction in interfacial energy caused by rare earth addition hinders the diffusion and growth of carbon atoms, effectively shortening their diffusion distance. Consequently, the pearlite interlamellar spacing in the RE-added steels 2 and 3 is finer than that in the RE-free sample 1 [34].

(1) $$\lambda =\frac{6\delta V}{\Delta G}$$

where λ is the spacing of pearlite lamellae; δ is the phase interface energy between carburizer and ferrite; *V* is the molar volume of pearlite; and Δ*G* is the free energy of phase transition.

### 3.2. Effect of Rare Earths La and Ce on the Mechanical Properties of U75V Steel

#### 3.2.1. Effect of Rare Earths La and Ce on the Hardness of U75V Steel

Hardness measures a material’s resistance to surface penetration by a harder object and is a key mechanical property of metals. It critically influences wheel-rail wear, noise generation, and safety in railway applications. Consequently, the Vickers hardness was measured for specimens from the three distinct experimental groups of U75V steel.

**Figure 13:**
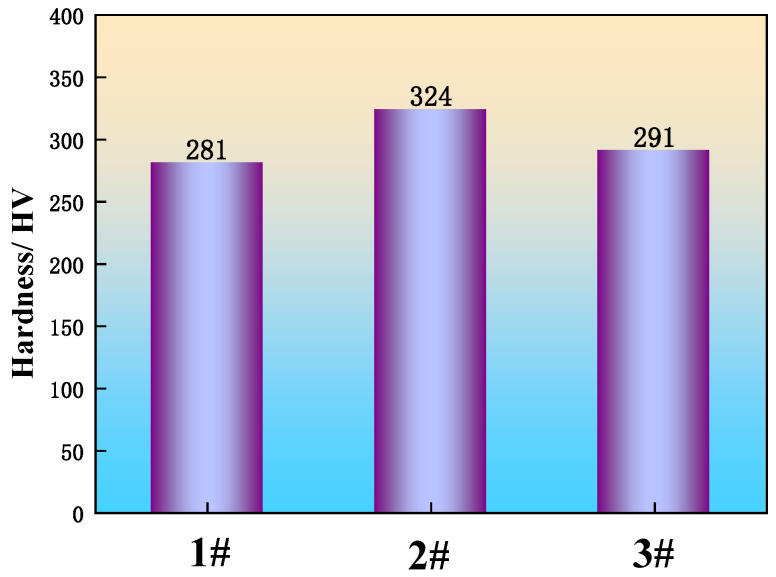
Hardness change in U75V steel.

To ensure the accuracy of the experiment, more than 10 sets of measurements were taken for each specimen, and the measurements were averaged; the results are shown in Figure 13. The hardness of sample 1 without added rare earths is 281 HV, and the hardness of samples 2 and 3 with added rare earths is 324 HV and 291 HV, respectively, which is an improvement of 15.3% and 3.6%, respectively. J. Maity et al. [35] developed a mathematical model correlating hardness with carbon content and microstructure in carbon steels. This model, which incorporates the effects of non-equilibrium eutectic carbon content and the Hall–Petch relationship, establishes a unique function relating the integrated hardness to the total carbon content, proeutectoid ferrite grain size, and pearlite interlamellar spacing. The expressions for the Vickers hardness of the ferrite and pearlite phases are given in Equations (2) and (3), respectively.

(2) $$H_{PF}=H_{PF}^{0}+K_{PF}D^{-0.5}$$

(3) $$H_{P}=H_{P}^{0}+K_{P}\lambda^{-0.5}$$

where $K_{PF}$, $H_{P}$ are constants specific to the system under consideration; *D* is the ferrite grain size in μm; and λ is the pearlite lamellar spacing in μm.

The cumulative effect of the hardness of a single microstructure on the total hardness of the eutectic steel can be considered by the multivariate rule, see Equation (4).

(4) $$H=f_{PF}H_{PF}+f_{P}H_{P}$$

where $f_{PF}$ is the volume fraction of ferrite organization, and $f_{P}$ is the volume fraction of pearlite organization.

The mathematical model developed by J. Maity et al. [35] was established by altering the steel microstructure through normalizing treatment to investigate its effect on hardness. The core of this model describes the quantitative relationship between microstructure and hardness. Given that U75V is a eutectoid steel, the model remains applicable to the U75V heavy rail steel in this study. According to Equations (2)−(4), the hardness of U75V steel is determined by the ferrite grain size and the pearlite interlamellar spacing. As a near-eutectoid steel with a carbon content of approximately 0.8%, U75V predominantly comprises pearlite, with a volume fraction significantly greater than that of ferrite. As evidenced by Figure 12, the pearlite interlamellar spacing in RE-added samples 2 and 3 is finer than in sample 1. Consequently, even considering the minor contribution of ferrite, Equations (3) and (4) predict that the Vickers hardness of the RE-added samples 2 and 3 should be higher than that of sample 1, which is consistent with the measured results.

#### 3.2.2. Effect of Rare Earths La and Ce on Tensile Properties of U75V Steel

**Figure 14:**
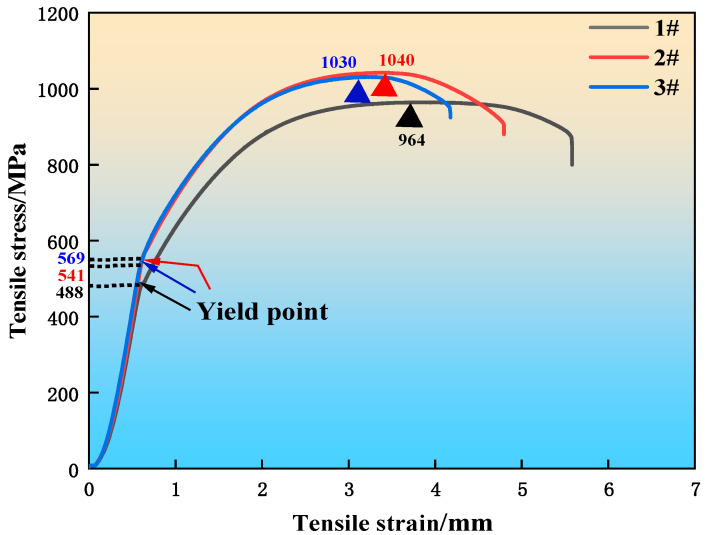
Tensile stress–strain curve of experimental steel.

Mechanical properties are critical performance indicators for heavy rail steel, with tensile strength and elongation after fracture being key metrics. Figure 14 presents the tensile stress–strain curves for the three experimental steel groups. All curves exhibit an initial elastic deformation stage, where no irreversible deformation occurs. Following the elastic stage, yielding begins, marked by the yield strength inflection point. Upon yielding, the curves plateau. Notably, the stresses for the REE-containing steels (samples 2 and 3) become significantly higher than that of the REE-free steel (sample 1). With continued loading and increasing deformation, the stress rises, but the rate of increase gradually diminishes until reaching the peak stress (tensile strength). Beyond this point, the stress decreases with increasing deformation until fracture occurs. According to Reference [36], the effective solubility limit of La in steel is approximately 0.018%. Exceeding this limit leads to a sharp rise in the volume fraction of inclusions, which in turn reduces material toughness. In sample 3, the total La content reaches 0.02%; this excess above the solubility limit resulted in decreased toughness.

**Figure 15:**
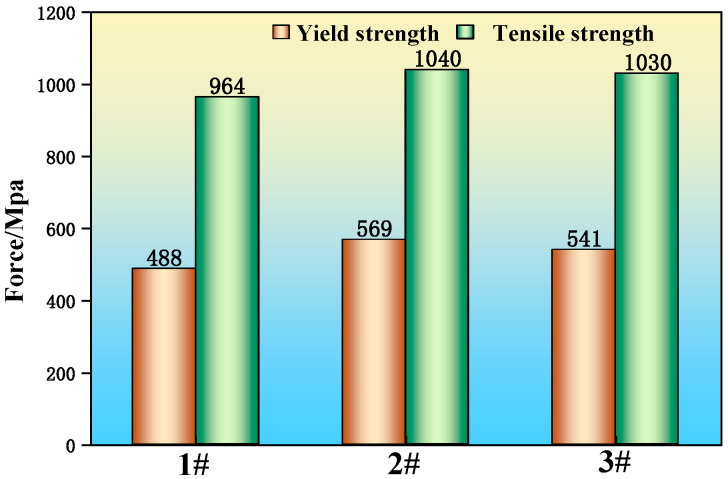
Yield and tensile strength of experimental steel.

Figure 15 shows the yield strength and tensile strength of the three experimental steels. The yield strength was 488 MPa for sample 1 (without RE addition) and 569 MPa and 541 MPa for samples 2 and 3 (with RE addition), representing increases of 16.6% and 10.9%, respectively. The tensile strength was 964 MPa for sample 1, and 1040 MPa and 1030 MPa for samples 2 and 3, corresponding to improvements of 7.9% and 6.8%, respectively. The improved strengths of the RE-added steels can be attributed to their refined microstructure. As previously shown, the addition of multi-component rare earths resulted in a smaller grain size and a finer pearlite interlamellar spacing in samples 2 and 3 compared to sample 1. Coarser microstructural features, such as larger grains and wider pearlite lamellae, are more prone to stress concentration from dislocation pile-ups. This facilitates plastic deformation in neighboring grains, thereby leading to the lower yield and tensile strengths observed in the RE-free sample 1.

Elongation at break is defined as the ratio of the elongation at fracture to the original gauge length in a tensile test. The reduction in area is defined as the percentage decrease in the cross-sectional area at the fracture relative to the original area. Both are key indicators of material toughness. The post-fracture elongation and reduction in area were measured using a micrometer. The results are summarized in Table 3 and Table 4. The original gauge length was 30 mm for all specimens. After testing, the lengths for samples 1 (no RE), 2, and 3 were 33.73 mm, 34.94 mm, and 33.95 mm, corresponding to elongations at break of 12.43%, 16.47%, and 13.16%, respectively. This represents an increase of 4.04 and 0.73 percentage points for samples 2 and 3 relative to sample 1. The original cross-sectional area was 19.625 mm^2^ for all specimens. The fracture surface areas for samples 1, 2, and 3 were 13.196 mm^2^, 11.94 mm^2^, and 12.876 mm^2^, resulting in area reductions of 32.76%, 39.16%, and 34.40%, respectively. These values correspond to an improvement of 6.40 and 1.64 percentage points for the RE-added samples 2 and 3 over sample 1.

**Figure 16:**
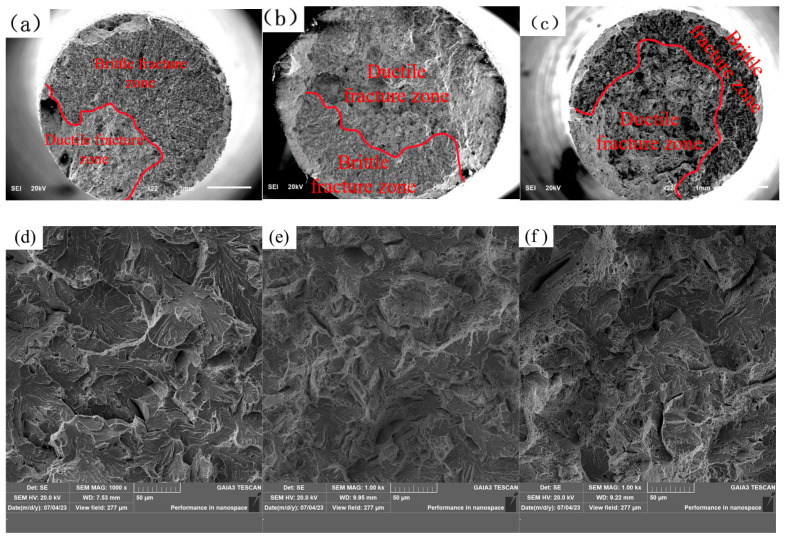
Tensile fracture morphology of experimental steel: (**a**) macro-morphology of sample 1; (**b**) macro-morphology of sample 2; (**c**) macro-morphology of sample 3; (**d**) micro-morphology of sample 1; (**e**) micro-morphology of sample 2; and (**f**) micro-morphology of sample 3.

Following the tensile tests, the fracture surfaces were kept clean and dry for subsequent analysis. The fracture morphology and associated inclusions were examined using scanning electron microscopy (SEM) to investigate the influence of multi-component rare earths (La and Ce) on the tensile properties of U75V steel from a microstructural perspective. The tensile fracture morphologies are shown in Figure 16, where panels (a–c) and (d–f) display the macro- and micro-morphologies, respectively. Macroscopic observation (Figure 16a–c) reveals that all three steels exhibited mixed brittle and ductile fracture modes. In sample 1 (without RE), the brittle fracture zone was larger than the ductile zone. In contrast, samples 2 and 3 (with RE addition) showed a smaller brittle zone compared to both their own ductile zones and the brittle zone of sample 1, indicating a distinct shift towards a more ductile fracture mode upon RE addition. Microscopic analysis of the brittle zones (Figure 16d–f) showed that sample 1 featured characteristic cleavage facets with river patterns, typical of brittle fracture. In comparison, the RE-added samples 2 and 3 exhibited a marked reduction in these river patterns and a significant increase in the population of fine dimples, indicative of enhanced micro-ductility.

**Figure 17:**
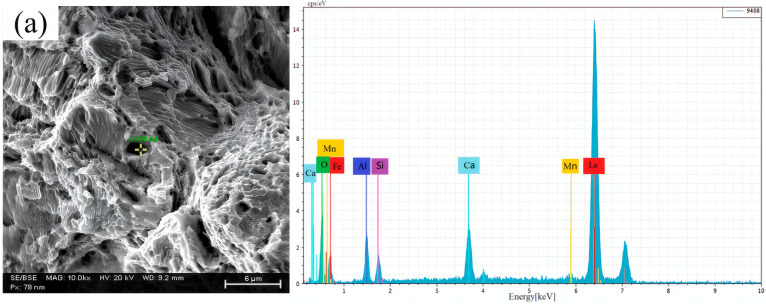

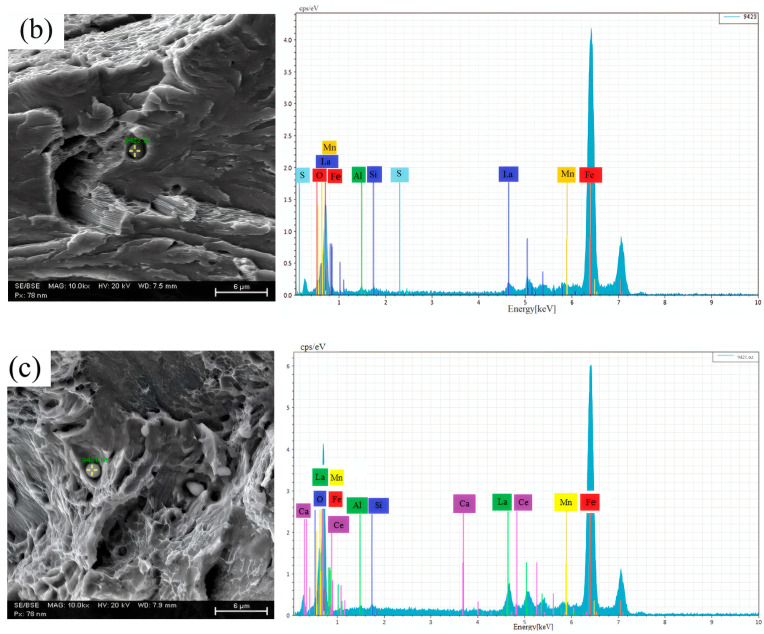
Microstructural analysis and energy-dispersive spectroscopy of inclusions at the fracture surfaces of tensile specimens from different experimental steels: (**a**) sample 1; (**b**) sample 2; (**c**) sample 3.

The inclusions on the tensile fracture surfaces were characterized using SEM. The marked area in the image indicates the location of the inclusion. The corresponding energy-dispersive spectroscopy (EDS) spectrum on the right shows the elemental composition analysis results of this inclusion, as shown in Figure 17. In sample 1 (without RE addition), the fracture surface contained Ca-Al-Si-O inclusions, which exhibited irregular shapes and were surrounded by large voids (Figure 17a). In contrast, the fracture surfaces of samples 2 and 3 (with RE addition) featured rare earth inclusions. These inclusions possessed regular spherical or ellipsoidal shapes and, unlike the Ca-Al-Si-O inclusions in sample 1, were not associated with peripheral voids (Figure 17b,c). This morphology indicates stronger interfacial bonding with the steel matrix, resulting in a less detrimental effect on the tensile properties.

#### 3.2.3. Effect of Rare Earths La and Ce on Impact Properties of U75V Steel

**Figure 18:**
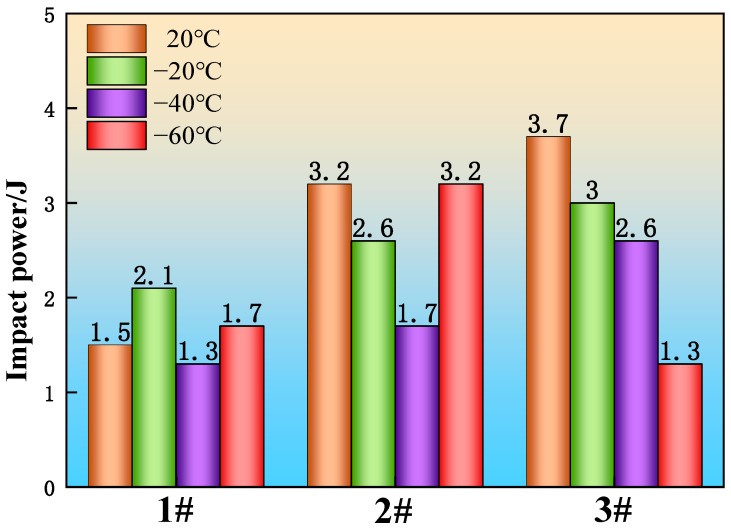
Impact energy of experimental steel at 20 °C, −20 °C, −40 °C, and −60 °C, respectively.

Considering the application of heavy rail steel in extremely cold northern regions, impact tests were conducted at 20 °C, −20 °C, −40 °C, and −60 °C on three groups of experimental steels to investigate the effects of adding La and Ce (multi-component rare earth elements) on the impact properties of U75V steel at ambient and low temperatures. As shown in Figure 18, the impact energy of sample 1 (without rare earth addition) was 1.5 J at 20 °C, while those of sample 2 and sample 3 (with La and Ce additions) were 3.2 J and 3.7 J, corresponding to increases of 113% and 146%, respectively. At −20 °C, the impact energy of sample 1 was 2.1 J, compared with 2.6 J and 3.0 J for sample 2 and sample 3, representing increases of 23.8% and 42.8%. At −40 °C, sample 1 exhibited an impact energy of 1.3 J, whereas sample 2 and sample 3 reached 1.7 J and 2.6 J, corresponding to improvements of 30.7% and 100%, respectively. At −60 °C, sample 1 showed an impact energy of 1.7 J; sample 2 reached 3.2 J (an 88.2% increase), while sample 3 dropped to 1.3 J, a decrease of 23.5% relative to sample 1.

Overall, the addition of multi-component rare earth elements improved the impact performance of the experimental steels at both room temperature and low temperatures, with the most pronounced enhancement observed at 20 °C. The only exception was sample 3, which exhibited reduced impact energy at −60 °C. At lower temperatures, the solubility of La in Fe is somewhat lower than that of Ce. Since the total La + Ce content in sample 3 exceeds that in sample 2, and the amount of rare earth elements in the steel surpasses their solubility limit, rare earth agglomeration occurs, leading to the formation of large-sized inclusions. These inclusions act as crack initiation sites under low-temperature impact conditions [37,38]. In general, the addition of rare earth elements refines and disperses inclusions in samples 2 and 3. These finer and more uniformly distributed inclusions effectively promote grain refinement, which helps to distribute stress across a greater number of grains when the material is subjected to external forces, thereby enhancing the impact toughness of the steel.

**Figure 19:**
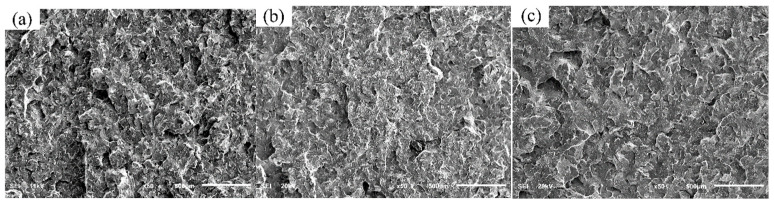

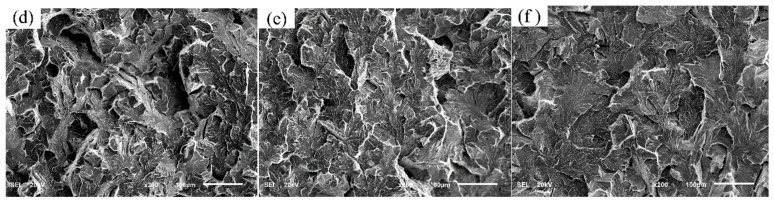
Normal temperature impact fracture morphology of experimental steel: (**a**) macro-morphology of sample 1; (**b**) macro-morphology of sample 2; (**c**) macro-morphology of sample 3; (**d**) micro-morphology of sample 1; (**e**) micro-morphology of sample 2; and (**f**) micro-morphology of sample 3.

Following the impact tests, the fracture surfaces were preserved clean and dry for subsequent analysis. The fracture morphology and inclusions were examined using scanning electron microscopy (SEM) to correlate the microstructural features with the impact properties. Figure 19 presents the fracture morphologies of the three steels after room-temperature impact testing, with Figure 19a–c and Figure 19d–f showing the macro- and micro-morphologies, respectively. Macroscopically, all three steels exhibited cleavage fracture surfaces, characteristic of brittle failure. As shown in Figure 19a, the fracture surface of sample 1 (without RE) was highly irregular with pronounced roughness. In contrast, the fractures of samples 2 and 3 (with RE addition) appeared comparatively smoother and flatter, as shown in Figure 19b,c. SEM observation at higher magnification revealed that sample 1 featured well-defined cleavage facets with distinct river patterns and large tear ridges (Figure 19d). Conversely, the RE-added samples 2 and 3 showed finer river patterns and a notably higher density of small dimples, indicative of localized micro-ductility, as shown in Figure 19e,f. These observations indicate that while all failures were predominantly brittle, the RE-added steels (samples 2 and 3) exhibited a clear trend towards a more ductile fracture mode, which correlates well with their superior impact energy. This enhancement can be attributed to grain refinement [38]. The finer grains in samples 2 and 3 increase the number of grain boundaries per unit volume, which hinders crack propagation, distributes plastic deformation more uniformly, and allows the material to absorb more energy before fracture.

**Figure 20:**
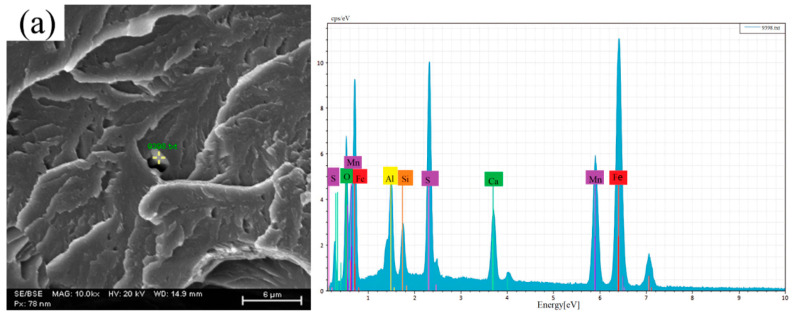

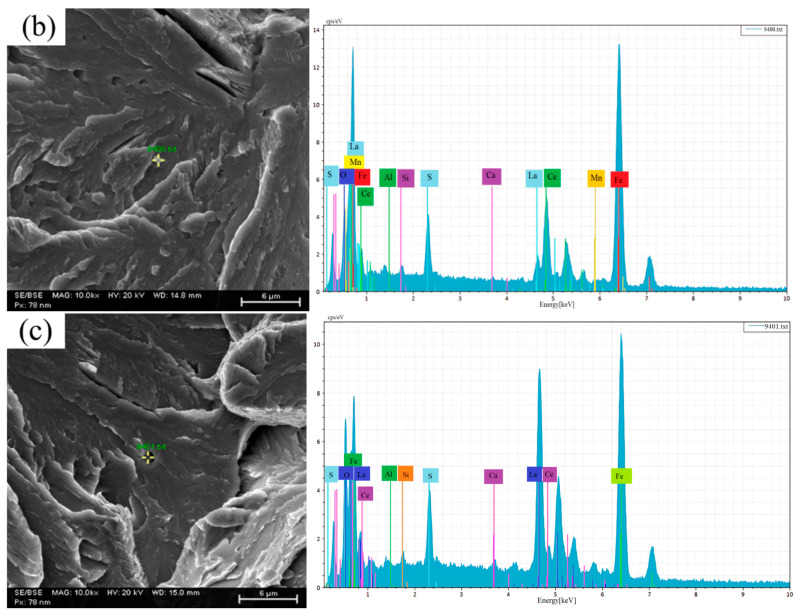
Microstructural analysis and energy-dispersive spectroscopy of inclusions at fracture surfaces of different experimental steel samples at room temperature: (**a**) sample 1; (**b**) sample 2; (**c**) sample 3.

The beneficial effect of rare earths on impact properties is also attributed to their modification of inclusions. Therefore, inclusions on the impact fracture surfaces were analyzed by SEM. The marked area in the image indicates the location of the inclusion. The corresponding energy-dispersive spectroscopy (EDS) spectrum on the right shows the elemental composition analysis results of this inclusion, with the results presented in Figure 20. In sample 1 (without RE), the impact fracture contained elongated composite inclusions comprising MnS and Ca-Al-Si-O (Figure 20a). Pronounced voids were present at the inclusion/matrix interface, compromising the microstructural integrity. These voids readily act as stress concentration sites and crack initiation points under external load, thereby degrading the impact resistance. In contrast, the fractures of samples 2 and 3 (with RE addition) contained RE_2_O_2_S inclusions. These inclusions were fine and spherical, with no evident voids at the interfaces (Figure 20b,c). Elongated MnS and Ca-Si-Al-Mn oxide inclusions, as shown previously in Figure 3, are highly deformable during hot forging. Such elongated inclusions can facilitate crack propagation along their length. The addition of rare earths modifies these inclusions into RE-containing types, which possess a stable, spherical morphology and are resistant to deformation. Furthermore, the thermal expansion coefficient of these rare earth inclusions is closer to that of the steel matrix. This reduced mismatch minimizes stress concentration under impact loading, effectively enhancing the impact performance across both room and low temperatures.

#### 3.2.4. Effect of Rare Earth La and Ce on the Wear Properties of U75V Steel

**Figure 21:**
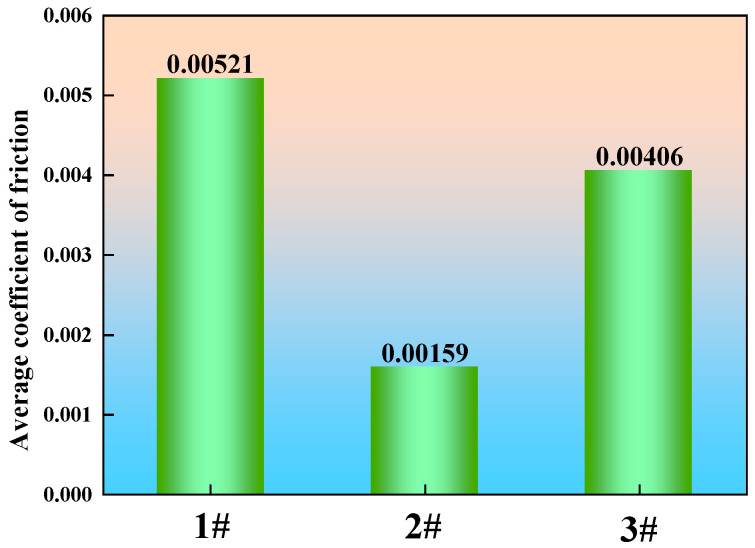
Average friction coefficients of the experimental steels.

The wear changes for the three sets of specimens are shown in Table 5. For the steel sample 1 without rare earth addition, the pre-wear weight was 0.266 N, and the post-wear weight was 0.258 N, with a wear amount of 0.008 N. Samples 2 and 3, containing multi-element rare earths, had pre-wear weights of 0.25 N and 0.249 N, respectively, with post-wear weights of 0.248 N and 0.248 N, resulting in wear amounts of 0.002 N and 0.001 N. This demonstrates that the wear amounts for steel samples 2 and 3, containing multi-element rare earths, are significantly lower than that of steel sample 1 without rare earths. During the wear test, the machine accurately measured the coefficient of friction to characterize the steel’s wear resistance. A lower average coefficient of friction indicates superior wear resistance of the material. The average friction coefficients of the three experimental steel groups are shown in Figure 21. The average friction coefficient of Steel No. 1 without rare earth addition was 0.00521, while those of samples 2 and 3 with multi-element rare earths were 0.00159 and 0.00406, respectively. Compared to sample 1 without rare earths, these values decreased by 69.5% and 22.1%.

## 4. Conclusions

Based on the systematic investigation into the effects of multi-element rare earths (La and Ce) on the microstructure and mechanical properties of U75V heavy rail steel, the main conclusions of this work can be summarized as follows:

(1) The inclusions in sample 1 (U75V steel without added rare earths) primarily consisted of MnS, oxide inclusions containing (Ca, Al, Si, Mn) elements, and composite MnS–oxide inclusions containing (Ca, Al, Si, Mn) elements. In contrast, the addition of mixed rare earths (La and Ce) in both sample 2 and sample 3 modified the original inclusions, resulting in the formation of RE2O2S inclusions and composite RE2O2S–oxide inclusions.
(2) The addition of rare earth elements significantly improved the purity and microstructure of U75V steel. Compared to the unreinforced sample 1 steel, the addition of rare earth elements in samples 2 and 3 steel resulted in a significant reduction in the number of inclusions, with an average size that was slightly smaller, demonstrating a notable purification effect. Additionally, rare earth elements effectively refined the microstructure—the average grain size of steel samples 2 and 3 decreased by 30.1% and 18.3%, respectively, and the average pearlite layer spacing also decreased by 32.5% and 37.5%, respectively.
(3) The enhancement of mechanical properties in steel through rare earth element addition does not follow a linear trend; instead, an optimal concentration range exists. Among the tested samples, sample 2 exhibited the most balanced improvement in overall performance. Specifically, compared to sample 1, sample 2 and sample 3 showed increases in hardness of 15.3% and 3.6%, respectively, and improvements in tensile strength of 7.9% and 6.8%. Meanwhile, their friction coefficients decreased significantly by 69.5% and 22.1%, respectively. Furthermore, rare earth addition improved impact toughness, with both samples 2 and 3 displaying higher impact energy than sample 1 at room temperature and in the low-to-medium temperature range. However, at −60 °C, the impact energy of sample 3 was 23.5% lower than that of sample 2. These results indicate that a moderate addition of rare earth elements contributes to comprehensive performance enhancement, whereas excessive addition adversely affects low-temperature toughness.

## Figures and Tables

**Table 1:** Technical parameter list of ZG 0.01 vacuum induction furnace.

Designation	Total Power of Equipment	Supply Voltage	Power Frequency	Medium Frequency Rated Voltage	Medium Frequency Rated Power	Rated Capacity	Rated Temperature
Parametric	50 kw	380 v	50 Hz	50 kw	370/250 v	0.01 t	1700 °C

**Table 2:** Chemical composition of experimental steel U75V (mass fraction/%).

Number	C	Si	Mn	V	S	P	Al	O	La	Ce
1#	0.73	0.63	0.87	0.062	0.002	0.0032	0.0016	0.0032	0	0
2#	0.72	0.65	0.94	0.061	0.0019	0.0045	0.0015	0.0021	0.0035	0.018
3#	0.76	0.57	0.88	0.056	0.0021	0.0036	0.0012	0.0028	0.02	0.0023

**Table 3:** Tensile elongation at break of experimental steels.

Number	Original Length/mm	Length After Tensile Fracture/mm	Elongation at Fracture
1#	30	33.73	12.43%
2#	30	34.94	16.47%
3#	30	33.95	13.16%

**Table 4:** Tensile section shrinkage of experimental steel.

Number	Original Cross-Sectional Area/mm^2^	Fracture Cross-Sectional Area/mm^2^	Shrinkage of Tensile Section
1#	19.625	13.196	32.76%
2#	19.625	11.94	39.16%
3#	19.625	12.876	34.4%

**Table 5:** Variation in wear of experimental steel.

Number	Weight Before Wear/N	Weight After Wear/N	Wear Amount/N
1#	0.266	0.258	0.008
2#	0.25	0.248	0.002
3#	0.249	0.248	0.001

## Data Availability

The original contributions presented in this study are included in the article. Further inquiries can be directed to the corresponding author.

## References

[1] Zhu H.Y., Yuan Y., Xiao Q., Li J., Zheng Y.X. (2021). Research Progress on Rail Corrugation. J. Traffic Transp. Eng..

[2] Zhou Y., Peng J.F., Zhao L., Wang W.J., Li W., Jin X.S., Zhu M.H. (2016). Damage Behavior of Wheel/Rail Materials Under Different Slip Rates. J. Mater. Eng..

[3] Ma L., Guo J., Liu Q.Y., Wang W.J. (2017). Fatigue Crack Growth and Damage Characteristics of High-Speed Rail at Low Ambient Temperature. Eng. Fail. Anal..

[4] Yang Z.Y., Zang J.J., Fang D.L., Li X., Li Z.Q., Li W.J. (2022). Thermal Fatigue Crack Propagation Mechanism of SiC_p_/A356 Composites for Urban Rail Train Brake Disc. J. Mater. Eng..

[5] Chen L., Wang H.J., Guo F.X. (2017). Effect of Quenching Microstructure on Fatigue Crack Growth Rate of Heavy Rail Steel. Mater. Rep..

[6] Li C.L. (2013). New Progress in the Application and Research of Rare Earths in Steel. Chin. Rare Earths.

[7] Fu X.Y., Yang J.C., Zhao L.P., Li M.D. (2015). The Action Mechanisms of RE Element in Steel and Its Study on Current Status. Hunan Nonferrous Met..

[8] Wang L.M., Du T., Lu X.L., Le K.N. (2001). Study of Behaviors and Application of Micro-Rare Earth Elements in Steel. Rare Earths.

[9] Guo Z.H., Liu E.K., Wang Q., Lou X.J., Liu H.B., Zheng Y.X., Wang B., Zhu L.G. (2023). Effect of Mg-Ce Treatment on Inclusion Characteristics and Pitting Corrosion Behavior in EH420 Marine Steel. Metals.

[10] Kang J., Yu Y.C., Liu L.G., Wang S.B., Chen W., Wen Y.B. (2021). Effect of Rare Earth on Inclusions and Impact Toughness of HRB500E Reinforcing Bar. J. Rare Earths.

[11] Guo H.F., Hao X. (2006). Effect of Rare Earth La on the Structure and Properties of 3Cr2W8V Hot Work Die Steel. Foundry Technol..

[12] Jie X.H., Li Z.Z., Yao T.G., Yu D.C. (1990). Effect of Rare Earth on the Thermal Fatigue Resistance of 5CrMnMo Steel. J. Heat Treat. Met..

[13] Nie Y.L. (2012). Influence of Ce-La on Purity, Microstructure and Performance of SS400 Steel. Master’s Thesis.

[14] Shi X.H., Yang L.L., Xia M., Xu Q.H., Zhao L.P. (2022). Modification effect of rare earth Ce content on inclusions in 4Cr5MoSiV1 steel. Heat Treat. Met..

[15] Fan L., Li C., Jiang M.F. (2020). Experimental Study on the Effect of Rare Earth on Inclusions in High-sulfur Free-cutting Steel. Iron Steel Vanadium Titan..

[16] Ma H.T., Wu D., Zhang Y.F. (2008). Rare Earth Adding Technology and Its Behavior in Steel for Heavy-Duty Truck Wheel. J. Rare Earths.

[17] Guo J., Li R., Zhang F. (2016). Research on Behavior Mechanism of Ce in Heavy Rail Steel. Heat Treat. Met..

[18] Yang J.C., Yang C.Q., Zhou L., Wang M.C. (2014). Effect of Cerium on Microstructures and Mechanical Properties of IF Steel. J. Rare Earths.

[19] Wu Y., Ma X., Wang P., Liu P., Wang J.J., Zhang K., Ma F.C., Li W. (2023). Research progress of function of rare earths in steel. Mater. Heat Treat..

[20] Yang Z., Zhao S., Xue Y.Q., Wang J.F., Sun X.L. (2024). Research progress on deformation behavior of clusions during billet rolling. J. Iron Steel Res..

[21] Zheng W., Wu Z.H., Li G.Q., Zhu C.Y. (2015). Effect of Rare Earth on Inclusions and Mechanical Properties of High-Strength Steel. Chin. J. Eng..

[22] Yang Z.Y., Wang M., Li Y.H. (2024). Research progress on influence and control of MnS inclusions on steel quality. J. Iron Steel Res..

[23] Zhu J., Huang H.Y., Xie J.X. (2017). Recent Progress and New Ideas for Accelerating Research in Rare Earth Steel. J. Iron Steel Res..

[24] Gui W.M., Liu Y., Zhang X.T., He L.L., Wang Y., Wang Y.D., He E.K., Wang M.M. (2021). Effect of Rare Earth Addition on Microstructure, Mechanical Property and Nitriding Performance of a Medium Carbon Steel. J. Mater. Res..

[25] Zhuo C., Liu R., Zhao Z.R., Zhang Y.L., Hao X.H., Wu H.J., Sun Y.H. (2022). Effect of Rare Earth Cerium Content on Manganese Sulfide in U75V Heavy Rail Steel. Metals.

[26] Hao W., Liu K., Zhu J., Ren L., Yang J.C. (2025). Influence of Rare Earth La–Ce on Inclusions and Impact Properties of U75V Steel. Metall. Res. Technol..

[27] Feng G.Q., Ren L., Yang J.C. (2024). Study on Influence of Rare Earth Ce on Micro and Macro Properties of U75V Steel. Materials.

[28] Zheng R.L., Tao Y. (2006). The Influence of Shape and Atomicity on the Surface Energy of Nanocrystal. Acta Phys. Sin..

[29] Tran R., Xu Z., Radhakrishnan B., Winston D., Sun W.H., Persson K.A., Ong S.P. (2016). Surface Energies of Elemental Crystals. Sci. Data.

[30] Zhang X.W., Zhang L.F., Yang W., Dong Y.C., Li Y.Z. (2016). Thermodynamics and Dynamics of Precipitation of MnS Inclusions During Solidification in Heavy-Rail Steels. Iron Steel.

[31] Bai G.J., Yang J.C., Liang W.J. (2023). Evaluation and Analysis of the Influence of Rare-Earth Ce on Inclusions in Heavy Rail Steel. Metals.

[32] Oleksak R.P., Detrois M., Jablonski P.D., Rozman K.A., Dogan O.N. (2024). Influence of Rare Earth Ce Additions on Microstructure and Mechanical Properties of Experimental Pipeline Steels. Steel Res. Int..

[33] Zhang H.S., Li J.H., Zhang D.P., Yue Y.Y., Yu S. (2023). Effect of rare earth La on microstructure and properties of Ferrium S53 ultra high strength steel. J. Iron Steel Res..

[34] Li X.K., Xin R.S., Yu Z.Y., Teng A.J., Kang J. (2024). Effect of Rare Earth Ce Content on Impact Properties of GCr15SiMn Bearing Steel. Bearing.

[35] Maity J., Poona B.S., Kumar M., Pal A., Hazra B., Sahin S., Biswas A. (2023). Quantitative hardness-carbon content-microstructure correlation in normalized plain carbon steel. Mater. Werkst..

[36] Wang C. (2017). Effect of Rare Earth Lanthanum and Magnesium on Cleanliness and Properties of Typical Special Steels. Ph.D. Thesis.

[37] Lü M., Gao X.Y., Xing L., Zhai T.T., Fang H.L., Wang H.Y., Wei H.H. (2025). Study on Solid Solution Behavior of Rare Earth Elements (La, Ce) in α-Fe Dilute Solution. Rare Earths.

[38] Fu Y. (2022). Effect of Rare Earth on Microstructure and Properties of Steel.

